# HSF1 at the crossroads of chemoresistance: from current insights to future horizons in cell death mechanisms

**DOI:** 10.3389/fcell.2024.1500880

**Published:** 2025-01-09

**Authors:** Shruti Ghai, Rejina Shrestha, Kuo-Hui Su

**Affiliations:** Department of Cell and Cancer Biology, College of Medicine and Life Sciences, The University of Toledo, Toledo, OH, United States

**Keywords:** heat shock factor 1, chemoresistance, proteotoxic stress response, autophagy, apoptosis, drug efflux

## Abstract

Heat Shock Factor 1 (HSF1) is a major transcriptional factor regulating the heat shock response and has become a potential target for overcoming cancer chemoresistance. This review comprehensively examines HSF1’s role in chemoresistance and its potential as a therapeutic target in cancer. We explore the complex, intricate mechanism that regulates the activation of HSF1, HSF1’s function in promoting resistance to chemotherapy, and the strategies used to manipulate HSF1 for therapeutic benefit. In addition, we discuss emerging research implicating HSF1’s roles in autophagy, apoptosis, DNA damage repair, drug efflux, and thus chemoresistance. This article highlights the significance of HSF1 in cancer chemoresistance and its potential as a target for enhancing cancer treatment efficacy.

## 1 Introduction

In 2024, roughly two million people are estimated to be diagnosed with cancer, with 611,720 cancer-related deaths in the United States (Siegel et al., 2024). Approximately 80%–90% of cancer-related deaths are attributed to the development of chemoresistance in responders (Ramos et al., 2021). Chemoresistance is responsible for most relapses, contributing to metastasis and poor rate of survival in patients (Ramos et al., 2021). Chemoresistance often leads to the need for more intensive chemotherapy regimens in second and later lines of cancer treatment as the initial therapies become less effective. The action of chemotherapies typically involves cellular uptake, intracellular activation, acting on the target site, and ultimately inducing cell death (Florea and Busselberg, 2011; Tilsed et al., 2022). Chemoresistance can occur at any of these stages, leading to failure of drug response. Cancers have been shown to develop resistance to various chemotherapies rapidly (Vasan et al., 2019; Ramos et al., 2021; Khan et al., 2024). For example, the resistance to the paclitaxel and 5-fluorouracil combination treatment was observed within 2 years since it being introduced to clinical use (Harris et al., 2006; Szakacs et al., 2006; Murray et al., 2012; Vulsteke et al., 2014). With the advancement in new targeted therapies and drug development, novel drugs show efficacy in many cancer types; however, these treated tumors often develop resistance to these drugs over time, making resistance a significant barrier to successful cancer treatment (Vasan et al., 2019; Khan et al., 2024). Hence, it is no surprise that chemoresistance contributes to about 80%–90% of cancer-related mortality.

Cancer cells utilize complex interplays between innate, intrinsic, and acquired factors to develop resistance to an administered drug during tumor development (Dzobo et al., 2018). In general, intrinsic chemoresistance is due to cell heterogeneity, whereas acquired resistance develops from chemotherapy-induced changes. The intrinsic chemoresistance of a tumor is evident when there is a lack of an initial response to the administered drug. The existence of intrinsic chemoresistance is largely due to the heterogeneity of the cancer cell population, especially the presence of cancer stem cells and mutations in key genes involved in cellular homeostasis and metabolism. These mutations can confer resistance by altering metabolic pathways, enhancing survival signaling, and impairing apoptotic response, thereby allowing cancer cells to survive and proliferate despite chemotherapy (Cancer Genome Atlas Research, 2014; Giordano, 2014; Hasan et al., 2018). Intrinsic chemoresistance is influenced by various factors, including the activation of signaling pathways like phosphoinositide 3-kinase (PI3K)/Akt, hedgehog, nuclear factor-κB (NFkB), and mitogen-activated protein kinase (MAPK), which have been shown to confer resistance to chemotherapeutic agents such as gemcitabine in pancreatic cancer (Rajabpour et al., 2017; He et al., 2021; Guo et al., 2024). The acquired chemoresistance only develops as a response to chemotherapy treatment and involves drug target mutations, tumor microenvironment modifications, and epigenetic changes such as methylation, acetylation, and microRNA (miRNA) expression survival (Mansoori et al., 2017; Emran et al., 2022). These alterations modulate upstream/downstream intracellular cell growth signaling such as MAPK and mammalian target of rapamycin (mTOR), modify the cell cycle checkpoints, inhibit apoptosis, and alter DNA replication, thereby enhancing cancer cell growth.

In addition to the mechanisms mentioned above that contribute to chemoresistance, accumulating evidence shows that stress response pathways are exploited by cancer cells to further support the chemoresistance processes (Vasan et al., 2019; Ramos et al., 2021; Khan et al., 2024). Stress responses, such as those triggered by oxidative stress, hypoxia, and DNA damage, initiate the survival and adaptive responses of cancer cells (Fulda et al., 2010; Dai et al., 2012; Chen and Xie, 2018). Stress responses also play crucial roles in the development and enhancement of resistance to chemotherapy. By inducting cytoprotective responses such as autophagy (Suh et al., 2012), the expression of heat shock proteins (HSPs) (Chatterjee and Burns, 2017), and the unfolded protein response, cancer cells can mitigate the effects of therapeutic stress, thereby promoting their continued growth and survival despite the treatment.

A better understanding of these chemoresistance mechanisms is essential for developing strategies to overcome drug resistance and improve the efficacy of cancer treatments. Recently, heat shock factor 1 (HSF1) has emerged as an intriguing player in tumorigenesis and chemoresistance (Alasady and Mendillo, 2020; Prince et al., 2020; Zhang et al., 2021). HSF1 is originally characterized as a transcription factor for the expression of HSPs responsible for the initiation of the proteotoxic stress response (Alasady and Mendillo, 2020; Zhang et al., 2021). This cellular mechanism safeguards cells from protein misfolding and aggregation under stress conditions (Alasady and Mendillo, 2020; Zhang et al., 2021; Ghai et al., 2024). HSF1 has since also been recognized for its pro-oncogenic properties, contributing to cancer initiation, progression, and chemoresistance (Kijima et al., 2019; Lee et al., 2021; Cyran and Zhitkovich, 2022; Gumilar et al., 2023).

The role of HSF1 in cancer has been extensively studied. HSF1 is downstream of the Kirsten rat sarcoma viral oncogene homolog (KRAS) signaling (Tang et al., 2015; Dai and Sampson, 2016). The *KRAS* gene is one of the most frequently mutated oncogenes in human cancers, with mutations commonly identified in approximately 90%–95% of pancreatic ductal adenocarcinoma (PDAC), 40% of colorectal cancers (CRC), and 25%–30% of non-small cell lung cancers (NSCLC) (Bailey et al., 2016; Zhu et al., 2021; Reita et al., 2022; Nusrat et al., 2024). Cancers with KRAS mutations are particularly lethal due to their role in promoting rapid cell proliferation and resulting in highly aggressive tumor phenotype (Huang et al., 2021). Recent research suggests that by regulating autophagy, apoptosis, DNA damage repair, or drug efflux mechanism, cancer cells can develop resistance to chemotherapy, thereby promoting tumor survival and progression (Dai and Sampson, 2016; Cyran and Zhitkovich, 2022; Gumilar et al., 2023). In this review, we will discuss the role of HSF1 in chemoresistance and its potential as a therapeutic target in tumorigenesis.

## 2 HSF1 in tumorigenesis

### 2.1 HSF1 is a key transcription factor in cancer development

**FIGURE 1:**
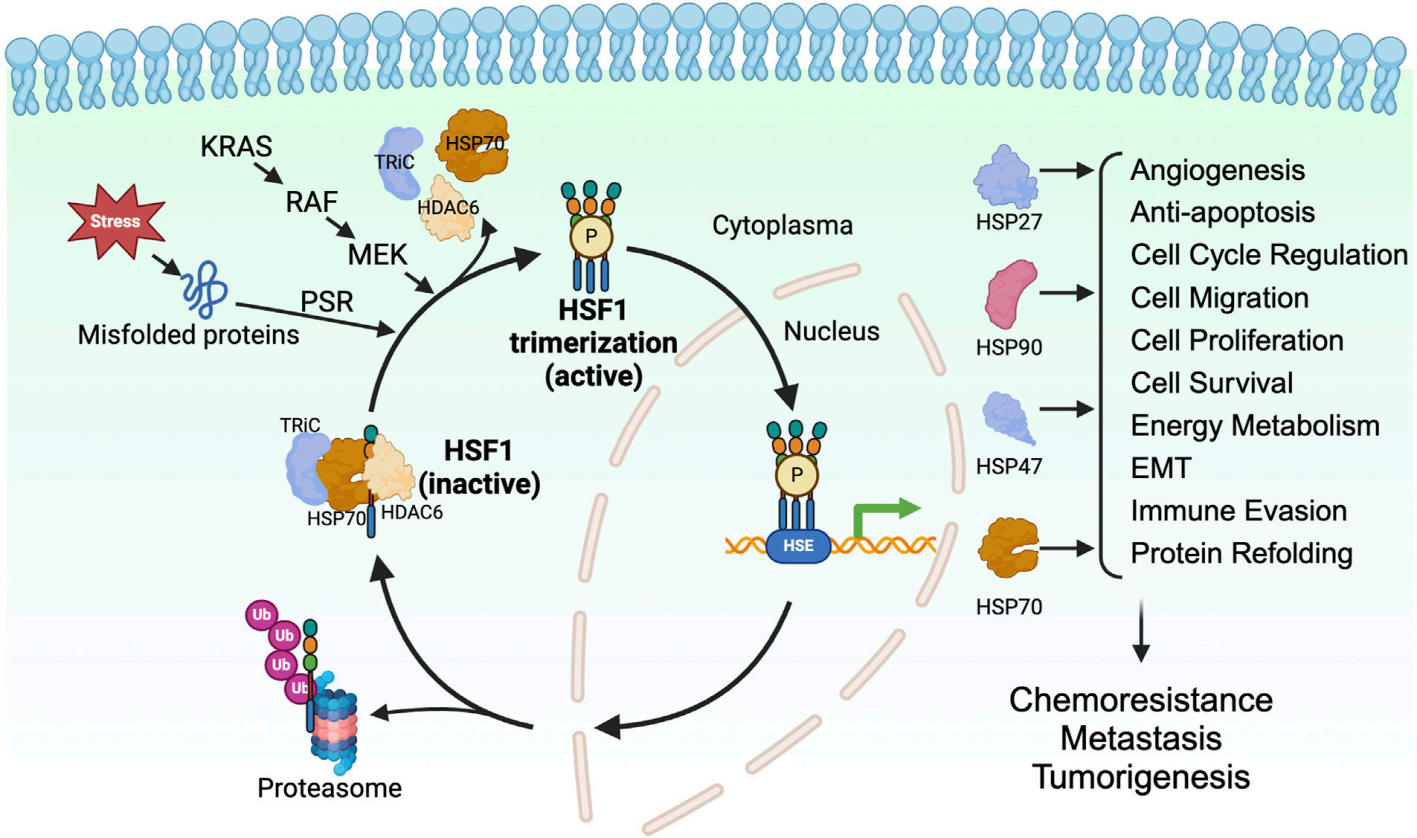
HSF1 is a transcriptional factor for HSPs in tumorigenesis. HSF1 is a downstream effector of KRAS-RAF-MEK signaling. HSF1 is activated upon proteotoxic stress in the cells, which allows its trimerization, phosphorylation, and translocation to the nucleus. In the nucleus, HSF1 binds to heat shock elements and allows expression of HSP27, HSP47, HSP70, and HSP90, which directly and indirectly affects tumor progression. EMT, epithelial-mesenchymal transition; HDAC6, histone deacetylase 6; HSE, heat shock element; HSF1, heat shock factor 1; HSP, heat shock protein; KRAS, Kirsten rat sarcoma viral oncogene homolog; MEK, mitogen-activated protein kinase kinase; P, phosphorylation; PSR, proteotoxic stress response; RAF, rapidly accelerated fibrosarcoma; TRiC, T-complex protein ring complex; Ub, ubiquitin.

When cells undergo proteotoxic stress from exposure to environmental factors such as heat shock, an adaptive cytoprotective mechanism, known as the heat shock response (HSR) or proteotoxic stress response (PSR), is activated. The HSR maintains the protein quality and prevents protein aggregation in the cells, ensuring the proteome homeostasis (proteostasis) in the cells (Lindquist, 1986). Immediately after the accumulation of unfolded proteins, HSF1, a master regulator of proteotoxic stress, is activated and, in turn, activates the transcription of genes encoding HSPs (Ghai et al., 2023). HSPs are molecular chaperones of the cells that enable the proper folding of the unfolded proteins and the degrading of the unrequired proteins through ubiquitination (Bukau et al., 2006; Gidalevitz et al., 2011; Dai and Sampson, 2016) (Figure 1).

HSF1 is a transcription factor and is an evolutionarily conserved member of the HSF family proteins (Wu, 1995; Anckar and Sistonen, 2011). HSF1 comprises the N-terminal helix-turn-helix DNA-binding domain, the oligomerization domain, and the C-terminal transactivation domain. The N-terminal DNA-binding domain of HSF1 binds to the inverted repeats of the heat shock elements (HSE) (Buchwalter et al., 2004). The oligomerization domain of HSF1 comprises four leucine zipper repeats, allowing HSF1 to trimerize and become an active transcription factor (Neudegger et al., 2016; Gomez-Pastor et al., 2018). The C-terminal transactivation domain of HSF1 is essential for the elongation process of transcription (Vihervaara et al., 2017). In cells under normal physiological conditions, HSF1 is present as a monomer and is sequestered by the HSP70, T-complex protein ring complex (TRiC), and histone deacetylase 6 (HDAC6) complex in the cytoplasm (Neef et al., 2014). Misfolded proteins resulting from proteotoxic stresses induced by various stimuli compete with HSF1 for binding to the chaperones, allowing the release of HSF1 from the HSP70/HDAC6 complex (Shi et al., 1998; Gomez-Pastor et al., 2018; Kijima et al., 2018). Along with the proteotoxic stress, post-translational modifications such as phosphorylation, SUMOylation, and acetylation are known to regulate the activation of HSF1. Phosphorylation of HSF1 at Ser326 is a commonly used marker for the activation of HSF1. In addition, phosphorylation of HSF1 on Ser230 and Ser320 positively regulates the HSF1 activity (Holmberg et al., 2001; Guettouche et al., 2005; Zhang et al., 2011; Chou et al., 2012a) whereas phosphorylation on Ser121 negatively regulates the activity of HSF1 (Dai et al., 2015; Swan and Sistonen, 2015). The acetylation of HSF1 on K298 prevents the proteosome-dependent degradation of the HSF1, thus increases the stability of HSF1 (Wu, 1995; Anckar and Sistonen, 2011). In contrast, the SUMOylation of HSF1 at K298 inhibits the activity of HSF1 (Wu, 1995; Hietakangas et al., 2003; Anckar and Sistonen, 2011). The activated HSF1 can trans-localize to the nucleus and regulate the transcription of genes encoding chaperone proteins. After the restoration of proteostasis, the HSP90 binds to the HSF1, leading to the inactivation of HSF1 (Gomez-Pastor et al., 2018; Kijima et al., 2018) (Figure 1).

Several studies using different murine models have shown HSF1 as a pro-oncogenic factor. A pioneering work performed in skin carcinogenesis mouse models shows reduced oncogenic Ras-induced tumor formation upon the loss of *Hsf1* (Dai et al., 2007). *Hsf1* deletion also leads to reduced tumor burden in other mouse models, including chemical carcinogenesis-driven hepatocellular carcinoma, mammary tumorigenesis resulting from p53 and neurofibromatosis type 1 (*Nf1*) loss, and lymphomas caused by p53-deficiency (Min et al., 2007; Jin et al., 2011; Xi et al., 2012). Along with that, increased HSF1 expression has been found in a wide range of human cancers, including cervix, colon, breast, lung, liver, pancreatic, and prostate carcinomas (Dudeja et al., 2011; Santagata et al., 2011; Fang et al., 2012; Mendillo et al., 2012). These reports indicate that HSF1 plays a crucial role in mediating tumorigenesis.

The increased rate of protein synthesis in cells allows for a high number of unfolded or misfolded proteins. Similarly, inflammation and stresses, such as hypoxia, activate HSF1 in the cells (Luo et al., 2009; Oromendia et al., 2012; Santagata et al., 2013). Besides this general activation of HSF1, cancer cells can constitutively activate HSF1, thus allowing its cytoprotective effect through various mechanisms. The mammalian target of rapamycin complex 1 (mTORC1) activated by PI3K/AKT phosphorylates HSF1 at Ser326 and accentuates the HSF1 transcriptional activity (Chou et al., 2012b). The HSF1 activation promotes cell proliferation while circumventing the cells' apoptosis and senescence (Meng et al., 2010; Carpenter et al., 2015). Besides, the Ras-MEK signaling phosphorylates HSF1 at Ser326 to activate HSF1’s transcriptional activity (Tang et al., 2015). In addition, the metabolic sensor AMP-activated protein kinase (AMPK) is reported to suppress the activity of HSF1 through phosphorylating the HSF1 Ser121 (Dai et al., 2015; Swan and Sistonen, 2015). In PDAC, the loss of AMPK allows the activation of HSF1, promoting the invasion and migration of PDAC (Chen et al., 2017). In summary, the activity of HSF1 is regulated by multiple upstream kinases and regulatory factors.

HSF1 activation in cancers thus is not only in response to different stresses but also through a wide array of other mechanisms, including those driven by oncogenic signaling and those that elevate HSF1 expression levels. This diverse range of HSF1 activation pathways collectively accounts for the extensive activation and functional roles of HSF1 in tumorigenesis.

### 2.2 HSF1-mediated upregulation of HSPs

Because HSF1 is a major transcriptional factor for HSPs, we will discuss the role of HSF1 in the chemoresistance mechanism. HSPs, including HSP70, HSP27, HSP90, HSP40, and HSP60, are crucial players in cancer chemoresistance (Zhang et al., 2021). These molecular chaperones facilitate protein folding, maintain protein stability, and, in some conditions, promote the degradation of misfolded or aggregated proteins, contributing to the maintenance of proteostasis within cancer cells. For example, HSP27 regulates the Salvador–Warts–Hippo pathway (Hippo pathway) known to control tumor progression and cancer stem cell reprogramming (Vahid et al., 2016). HSP27 forms multimeric complexes to stabilize denatured and aggregated proteins, making them functional (Vahid et al., 2016). HSP40 aids proper protein folding, translation, translocation, and degradation (Takashima et al., 2018). Several HSP40 family members are highly expressed in various types of human cancer, including colorectal, gastric, and KRAS-mutated lung cancers. Concerning colorectal cancer, HSP40 has demonstrated metastatic promoting behavior (Sterrenberg et al., 2011; He et al., 2015; Yamashita et al., 2016; Yang et al., 2020). Elevated levels of HSPs can promote chemoresistance by aiding the proper folding of oncoproteins, thereby sustaining malignant processes. HSP90 is overexpressed in multiple cancer types and regulates the stability, activity, maturation, and proteolytic degradation of various oncogenic kinases. HSP90 interacts with its substrates through its N-terminal ATPase domain, which is enhanced by the binding of co-chaperones such as HSP70 (Chiosis et al., 2004; Workman et al., 2007; Chatterjee et al., 2016).

HSPs also directly regulate kinases. For example, overexpressed HSP90AA1 upon chemotherapy dissociates phosphorylated AKT and c-Jun N-terminal kinase (JNK), inducing protective autophagy while inhibiting apoptosis and hence contributing to chemoresistance (Xiao et al., 2018). This highlights the important role of HSPs in chemoresistance and underscores how highly regulated cellular pathways are hijacked by cancer cells to survive stress and chemotherapy. Furthermore, cancer cells exploit external signaling by binding extracellular HSP90α to lipoprotein receptor-related protein 1 (LRP1), which has been shown to promote PDAC metastasis via AKT activation. LRP1 is associated with poor PDAC patient survival and LRP1 silencing increases the susceptibility of PDAC cells to doxorubicin and gemcitabine (Xue et al., 2022). Disruption of HSP47 in PDAC cells increases intracellular reactive oxygen species (ROS) and subsequent Ca^2+^ levels, resulting in the activation of the caspase-12/caspase-9/caspase-3 axis, which may sensitize cells to chemotherapy (Yoneda et al., 2021). Inhibition of HSP27 in human colon cancer cells reduces their acquired resistance to 5-fluorouracil (Asada et al., 2021). This suggests that HSPs play a role in protecting cells from ROS-induced damage and cellular stress. There is still a significant gap in our understanding of how HSPs modulate oxidative stress and provide protection against ROS, further investigation into these mechanisms could shed light on their contributions to chemoresistance.

In summary, HSF1-mediated regulation of HSPs provides diverse mechanisms of chemoresistance by preserving proteomic integrity and modulating cell death pathways. The upregulation of HSPs through HSF1 thus promotes tumor growth and contributes to chemoresistance (Figure 1). Given this understanding, targeting HSF1 in various cancers appears to be a promising therapeutic strategy. We will further discuss the role of HSF1 in chemotherapy and its effect on chemoresistance. The combination of HSF1 inhibition with existing chemotherapeutic agents may represent a potential avenue for enhancing treatment efficacy and warrants further investigation. We have summarized recent studies and the current mechanisms on the role of HSF1 in driving chemoresistance, which may provide new avenues for targeting HSF1 in various cancers.

## 3 HSF1 as a regulator of chemoresistance

### 3.1 HSF1 and autophagy

PSR regulates both the ubiquitin-proteasome system (UPS) and autophagy for the degradation of dysfunctional and toxic proteins to maintain proteostasis in response to stress. HSF1 induces autophagy by acting as a transcription factor of multiple autophagy-related genes (ATG), such as ATG5, ATG7, and ATG12. HSF1 also induces the autophagy marker sequestosome 1 (SQSTM1)/p62 activity through the regulation of its upstream kinases (Watanabe et al., 2017). However, the autophagy flux and the p62 level are increased in HSF1-deficient mice (Dayalan Naidu et al., 2017). This suggests that the relationship between HSF1 and autophagy may be context dependent.

In the past decade, various studies have demonstrated that autophagy plays an important role in conferring chemoresistance. ATGs, such as ATG3, ATG5, ATG6 (Beclin-1), ATG7, and ATG12, and autophagy markers, such as microtubule-associated protein 1A/1B-light chain 3 (LC3) and p62, are the major regulators of autophagy and chemoresistance (Li et al., 2019). ATG3 facilitates the conversion of LC3-I to LC3-II and promotes endoplasmic reticulum (ER) stress. However, the downregulation of ATG3 increases the sensitivity to erlotinib in the erlotinib-resistant lung cancer cell lines (Lee and Wu, 2012). ATG5 normally participates in the elongation of the autophagosome membrane. However, the interaction of the long non-coding RNA (lncRNA) gallbladder cancer drug resistance-associated lncRNA1 with phosphoglycerate kinase 1 in gallbladder cancer cells prevents ATG5 degradation and induces the ATG5-ATG12 complex formation, promoting autophagy and doxorubicin resistance (Cai et al., 2019). Additionally, suppression of ATG6, a crucial protein in the ATG6 and Vps-34 complex that promotes autophagosome formation, downregulates HER2 expression and enhances tamoxifen sensitivity in estrogen receptor-positive breast cancer cells *in vitro* (Gu et al., 2017). ATG7, an activator of ATG8 involved in the expansion of phagophore, confers chemoresistance in the acute myeloid leukemia cell lines against cytarabine and idarubicin treatment, knockdown of ATG7 increased apoptosis and DNA damage (Piya et al., 2016). Hence, autophagy is critical in chemoresistance, involving multiple autophagy-related genes and proteins (Debnath et al., 2023; Mohammed et al., 2024). This observation supports the well-established phenomenon of autophagy functioning in an oncogenic manner, contingent upon the stage of tumorigenesis. Autophagy is cytoprotective under stress conditions, such as chemotherapy, and helps maintaining the cellular homeostasis in the surviving cancer cells, such as colorectal and hepatocellular carcinoma cells (Xu et al., 2013; Lan et al., 2024). Therefore, monitoring the stages of tumorigenesis and autophagy and employing autophagy inhibitors may open new therapeutic avenues to combat chemoresistance.

**FIGURE 2:**
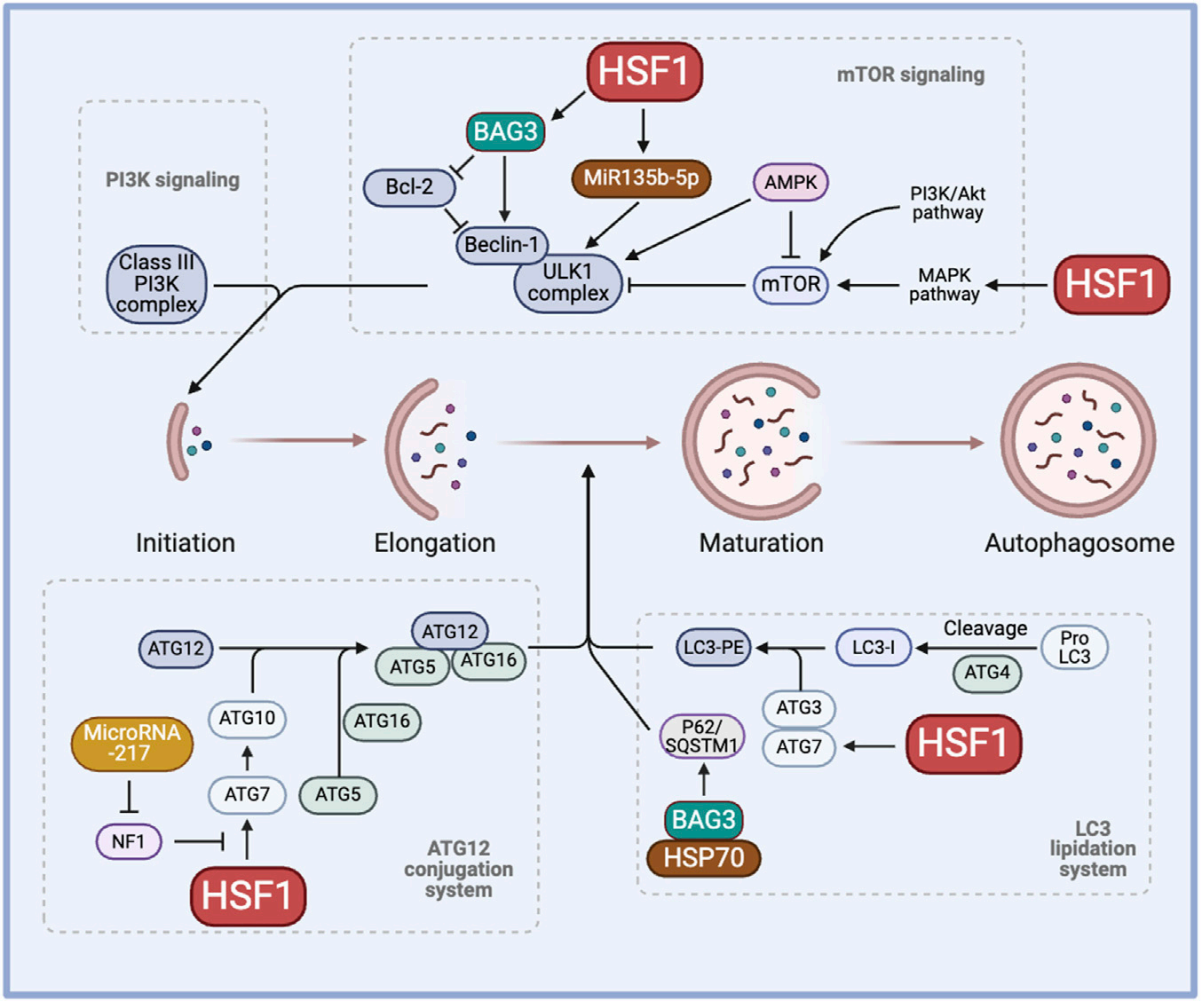
The role of HSF1 in cancer autophagy. HSF1 is involved in cancer autophagy by regulating the initiation process through the PI3K-mTOR signaling, the elongation process through the ATG12 conjunction system, and the maturation of autophagosome formation through the LC3 lipidation system. This regulation induces cytoprotective autophagy, hence regulating chemoresistance. AMPK, AMP-activated protein kinase; ATG, autophagy-related genes/proteins; BAG3, Bcl2-associated athanogene 3; HSP, heat shock proteins; HSF1, heat shock factor 1; LC3, microtubule-associated protein 1 light chain 3; LC3-PE, LC3-phosphatidylethanolamine conjugate; MAPK, mitogen-activated protein kinase; miR, micro RNA, mTOR, mammalian target of rapamycin; NF1, neurofibromin 1; PI3K, phosphoinositide 3-kinase; SQSTM1, sequestosome 1/p62; ULK1, Unc-51 like autophagy activating kinase 1.

HSF1 is correlated with various ATGs and their activity. Higher levels of nuclear HSF1 result in a higher H3 acetylation level and enhanced activity of the ATG7 promoter. The absence of HSF1 prevents the ATG7 promoter activity and increases the chemosensitivity to carboplatin in the MDA-MB-231 breast cancer cell line (Desai et al., 2013). Thus, inhibiting cytoprotective autophagy induced by HSF1 knockdown could provide a better therapeutic efficacy against drug resistance. In a study showing that BAG3 contributes to chemoresistance, 5-Fluorouracil and Doxorubicin-resistant triple negative breast cancer cell lines show higher expression of ATG5, LC3-II, and Beclin-1, suggesting a correlation between drug resistance and autophagy (Das et al., 2018). Importantly, inhibiting the transcriptional activity of HSF1 significantly increases the sensitivity of these resistant cell lines to the treatment. Bcl2-associated athanogene 3 (BAG3) also works together with the molecular chaperones HSP70 and HSPB8 along with the ubiquitin receptor SQSTM1/p62 to selectively direct aggregation-prone proteins for degradation via autophagy (Minoia et al., 2014; Sturner and Behl, 2017). Moreover, transmission electron microscopy revealed a distinct accumulation of small vacuoles in the cytoplasm of cells expressing BAG3, indicating enhanced autophagic flux, in HepG2 and MCF7 cells. The enhanced autophagic flux is further supported by a significant increase in LC3-II and p62 levels (Zhao et al., 2019). These studies highlight the novel HSF1-BAG3 axis, targeting which may pave the way to increase chemosensitivity to current therapies in cancer (Figure 2).

HSF1 has been shown to induce miR-135b-5p overexpression, which induces protective autophagy, in colorectal cancer following oxaliplatin treatment. MiR135b-5p stabilizes Unc-51 like autophagy activating kinase 1 (ULK1) by inhibiting mitochondrial E3 ubiquitin protein ligase 1 and its E3 ubiquitin ligase activity on ULK1, thereby inducing protective autophagy and resistance against oxaliplatin (Wang et al., 2021). MiR-217 is known to regulate the HSF1-ATG7 axis by inhibiting the NF1 activity and enhancing chemoresistance (Li et al., 2023a). Other microRNAs, such as miR-107, have been shown to inhibit autophagy and decrease breast cancer progression by targeting HMGB1. Therefore, overexpressing miR-107 can serve as a strategy to hinder cancer progression whereas inhibiting miR-217 or miR-135b-5p may aid in regulating autophagy to combat drug resistance (Ai et al., 2019). Given the diverse roles of microRNAs across various cancers and stress factors, it is crucial to adopt a more mechanistic perspective to comprehend the clinical significance of microRNAs in chemoresistance (Figure 2).

The role of HSF1 and HSPs in tumor autophagy remains a topic of ongoing debate. HSF1 promotes autophagy by upregulating ATG10 through binding to the *Atg10* promoter, thereby enhancing the lipidation of LC3-II. In contrast, HSF1 depletion reduces ATG10 expression and increases the production of inflammatory cytokines in lipopolysaccharide-treated peritoneal macrophages (Tan et al., 2023). Manganese exposure induces hepatic mitochondrial oxidative stress, leading to HSF1 phosphorylation at Ser326 and activation of autophagy. Knockdown of HSF1 prevents manganese-induced autophagosome formation in hepatocytes of yellow catfish (Zhao et al., 2024). Conversely, HSF1 regulates JNK1-mediated mTORC1 activation, suggesting an inhibitory role in autophagy (Su et al., 2016; Su and Dai, 2016; Su and Dai, 2017). Furthermore, HSF1 knockdown activates AMPK and promotes mitophagy, leading to reduced mitochondrial mass (Su et al., 2019). Moreover, recent studies show that HSP70 negatively regulates autophagy and that HSP70 inhibition, along with autophagy blockade, promotes cell death in NSCLC cells (Alhasan et al., 2024). These findings demonstrate the complexity of HSF1 in autophagy, highlighting the need for further investigation to clarify its functions in autophagy regulation and its contribution to chemotherapy resistance (Figure 2).

Future research should focus on clarifying the dual role of HSF1 in autophagy regulation, investigating the specific conditions and signaling pathways that determine whether HSF1 acts as a promoter or inhibitor of autophagy. Additionally, exploring the impact of HSF1-mediated autophagy on chemoresistance in cancer could reveal new strategies to enhance the therapeutic sensitivity in autophagy-dependent tumors. Finally, studying the interactions between HSF1, HSP70, and key pathways, such as kinase activities and ATG protein expression, may provide insights into combination therapies targeting autophagy and chemoresistance in cancer treatment.

### 3.2 HSF1 and apoptosis

Both intrinsic and extrinsic pathways of apoptosis contribute to chemoresistance. The involvement of the intrinsic pathway is indicated by the upregulation of anti-apoptotic proteins, such as BCL-2 family proteins, and the downregulation of proapoptotic proteins, such as BAX and BAK (Singh et al., 2019). Decreased expression and increased endocytosis of molecules involved in the extrinsic pathway, such as tumor necrosis factor superfamily proteins, FAS ligands, and death receptor (DR) 4 and DR5, potentially confer drug resistance (Ashkenazi, 2015).

HSF1 primarily functions as a transcription factor of HSP70 and HSP90, which are well-known for their roles in inhibiting apoptosis. For instance, HSP70 plays a protective role in heat-induced apoptosis by stabilizing the anti-apoptotic protein MCL-1, which prevents BAX activation and cytochrome-c release (Stankiewicz et al., 2009). Similarly, HSP90 prevents the release of cytochrome c by inhibiting the activity of the proapoptotic protein FKBP38. Increased HSP90 expression is correlated with elevated levels of antiapoptotic proteins BCL-2 and BCL-xL (Edlich et al., 2007). On the other hand, inhibiting HSP27 triggers the release of SMAC protein, a key regulator of the mitochondrial apoptotic pathway, in dexamethasone-resistant myeloma cell lines and promotes the activation of caspase-9 and caspase-3, suggesting that HSP27 contributes to resistance against dexamethasone (Chauhan et al., 2003).

Moreover, genetic knockdown of HSF1 has been shown to directly link to enhancing apoptosis through the regulation of the mitochondrial apoptosis pathway. In breast cancer, HSF1 knockdown enhances BAX expression and cisplatin-induced apoptosis, whereas restoring HSF1 expression significantly reduces cisplatin-induced apoptosis (Liu and Ma, 2021). In a study on pancreatic tumorigenesis, HSF1 silencing led to the upregulation of pro-apoptotic proteins, including SMAC, cytochrome c, Apaf1, and cleaved caspase-3 and -9, suggesting that HSF1 inhibits the mitochondrial apoptosis pathway to promote tumor growth (Liang et al., 2017). Inhibiting HSP70 prevents HSP70 from stabilizing anti-apoptotic proteins and blocking apoptosis, thus reducing chemoresistance, in bladder cancer (Wei et al., 2024). Inhibiting HSF1 by small molecules enhances the effectiveness of the aurora kinase inhibitor efficacy in NSCLC by promoting apoptosis, potentially overcoming chemoresistance through PI3K/AKT pathway downregulation and ROS activation (Zhang et al., 2024). This suggests that inhibiting HSF1 enhances the effectiveness of chemotherapy by overcoming HSF1- or HSP70-mediated anti-apoptosis mechanisms.

**FIGURE 3:**
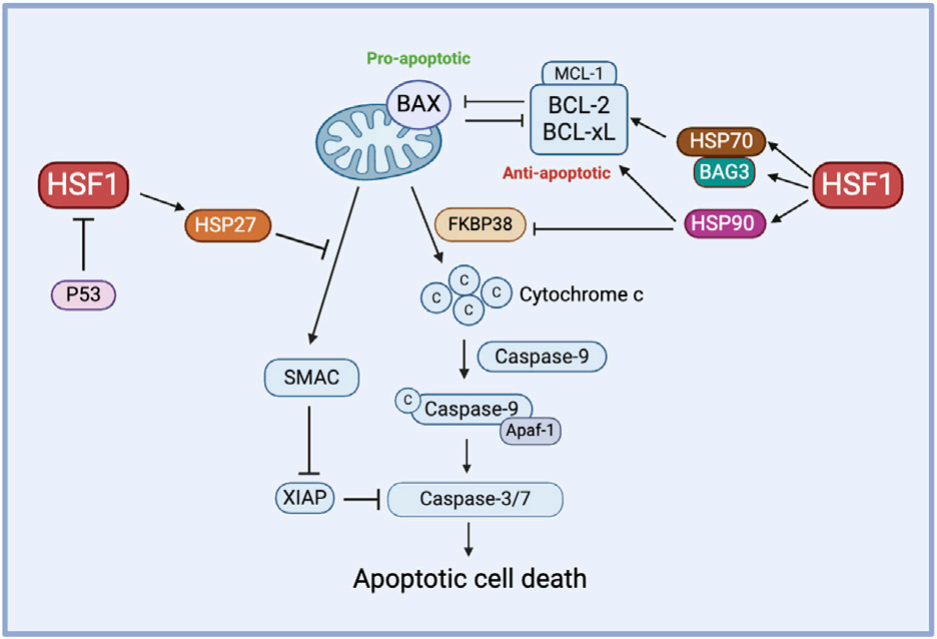
The role of HSF1 in cancer apoptosis. HSF1 mediates pro-apoptotic and anti-apoptotic pathways in the chemoresistance by regulating the expression of downstream HSPs. HSP70 controls the function of anti-apoptotic proteins via the HSP70-BAG3 axis, HSP90 regulates anti-apoptotic proteins and inhibits FKBP38, and HSP27 prevents the release of SMAC. Additionally, P53 inhibits HSF1 to avert this process of apoptosis. Apaf-1, apoptotic protease activating factor 1; BAG3, Bcl2-associated athanogene 3; BAX, Bcl-2-associated X protein; BCL-xL, B-cell lymphoma-extra large; c, Cytochrome c; FKBP38, FK506-binding protein 38; HSF1, heat shock factor 1; HSP, heat shock protein; MCL-1, myeloid cell leukemia-1; P53, tumor protein p53; SMAC, second mitochondria-derived activator of caspases; XIAP, X-linked inhibitor of apoptosis protein.

BAG3, a member of the co-chaperone family, is a well-known non-HSP substrate of HSF1 and contributes to chemoresistance in cancer (Jacobs and Marnett, 2009; Antonietti et al., 2017; Guo et al., 2022). Generally, HSP70’s function depends on its interactions with other chaperones like HSP90 and the co-chaperone BAG3. In an interesting study, the HSF1/HSP70/BAG3 pathway is investigated and confirmed to contribute to chemoresistance, particularly through its role in the overexpression of pro-survival BCL-2 family proteins and the subsequent resistance to cell death in gliomas (Das et al., 2018). In another study, the overexpression of Bag-1, another protein from the BAG family, modulates HSP levels by phosphorylating HSF1 at Ser326 via the PI3K/AKT/mTOR pathway in HER2-positive and HER2-negative breast cancer cells. BAG1 explicitly enhances the expression of HSP70 and HSP27, contributing to breast cancer cell survival and, potentially, drug resistance (Kizilboga et al., 2024). HSF1 influences apoptosis and chemoresistance through its interactions with co-chaperones like BAG3 and BAG1 and it promotes cell survival by upregulating pro-survival BCL-2 family proteins and HSPs across various cancer types (Figure 3).

Other stress response mechanisms also contribute to the HSF1-mediated chemoresistance. For example, ER stress-induced HSF1 promotes chemoresistance to ubiquitin-specific protease 7 (USP7) inhibitors via the protein kinase R-like ER kinase (PERK) pathway, suggesting that targeting HSF1 or PERK could improve USP7 inhibitor-based chemotherapy (Lim et al., 2024). Besides, The Munc18-1 interacting protein 3- (Mint3)-activated hypoxia-indued factor 1α (HIF-1α) signaling promotes chemoresistance in triple-negative breast cancer (TNBC) by increasing the HSP70 expression. Mint3 depletion induces energy stress, which inactivates HSF1 via the AMPK/mTOR pathway, reducing HSP70 levels and enhancing the effectiveness of doxorubicin in TNBC (Tanaka et al., 2023). These findings suggest crosstalk among stress response signaling pathways, with HSF1 driving chemoresistance through mechanisms like ER stress via PERK and HIF-1α signaling, ultimately enhancing cancer cell survival during chemotherapy.

In addition, the interaction of HSF1 with multiple oncogenic transcription factors is crucial for tumorigenesis and apoptosis. The wildtype p53 is a tumor suppressor, inducing cell cycle arrest and apoptosis when DNA damage cannot be repaired. Surprisingly, mutant p53 directly interacts with HSF1, aiding the proper binding of HSF1 to the target HSPs and regulating the activity of HSPs, thereby contributing to cell survival. Moreover, mutant p53 is also known to activate mTOR, MAPK, and PI3K pathways via erythroblastic oncogene B (ErbB) family, including epidermal growth factor receptor (EGFR) and HER2, inducing HSF1 activation and contributing to apoptosis and chemoresistance (Toma-Jonik et al., 2019; Zhang et al., 2021). Furthermore, SUMOylation of HSF1 at the K298 enhances its stability, nuclear localization, and mitochondrial unfolded protein response, which promotes glioblastoma cell proliferation, migration, and resistance to apoptosis (Li et al., 2024). This SUMOylation-modified HSF1 activity contributes to chemoresistance by supporting mitochondrial function and increasing the expression of mitochondrial chaperones, which may help cancer cells to evade chemotherapy-induced stress. Future research should explore how targeting HSF1 and its downstream pathways can be used to overcome chemoresistance, focusing on both intrinsic and extrinsic apoptotic mechanisms and the HSF1’s effect on other programmed cell death signaling.

### 3.3 HSF1 and DNA damage repair

The DNA damage response (DDR) is a complex network of pathways that maintain the genome integrity. A key mechanism of DDR is base excision repair, which is mediated by Poly (ADP-ribose) polymerase (PARP) and AP endonuclease1 (APE1) as the responsible proteins. Other pathways contributing to DDR include non-homologous end joining (NHEJ) and homologous recombination (HR). DNA–dependent protein kinase (DNA-PK) plays an important role in NHEJ for double-strand breaks (DSBs) and contributes to chemo-radiotherapy resistance. DSBs are critical DNA lesions repair mainly by HR or NHEJ. Ataxia-telangiectasia mutated and ataxia telangiectasia and Rad3-related (ATR) kinases detect DSBs, activating p53 for cell cycle arrest or apoptosis and Breast cancer genes (BRCA) 1/2 for accurate repair through HR (Jackson and Bartek, 2009). This coordinated response maintains genomic stability, preventing mutations that can lead to cancer. Cell cycle checkpoint kinases are activated during DDR, of which checkpoint kinase 1 (CHK1) and CHK2 are downstream substrates of ataxia-telangiectasia mutated (ATM)/ATR. ATR and ATM are activated during single-strand and double-strand breaks, respectively. The enhanced DNA damage repair capacity (DRC) in tumor cells contributes significantly to their drug resistance, including targeted and immune therapy For example, cisplatin-resistant tumor cells often demonstrate higher expression of DNA damage repair-related genes and higher DRC (Fujimoto et al., 2017). Furthermore, inhibiting the nucleotide excision repair (NER) pathway further increased the sensitivity of tumor cells to another chemotherapy drug, cisplatin, complementing the cytotoxic effects (Oliver et al., 2010; Wang et al., 2011). HSF1 plays a crucial role in DNA damage repair-mediated chemoresistance, particularly through its involvement in the NHEJ pathway. HSF1 inhibits NHEJ by interacting with Lupus Ku autoantigen protein p70 (Ku70) and Ku86 to disrupt their heterodimeric interaction, leading to defective DNA repair and genomic instability. This is a potential mechanism for HSF1-mediated carcinogenesis (Kang et al., 2015). This inhibition of NHEJ by HSF1 contributes to chemoresistance by promoting genomic instability, which can drive cancer progression and resistance to therapy. Notably, HSF1’s function in NHEJ appears to be independent of its traditional role as a transcription factor, it instead operates through direct protein-protein interactions within the DNA damage repair machinery.

**FIGURE 4:**
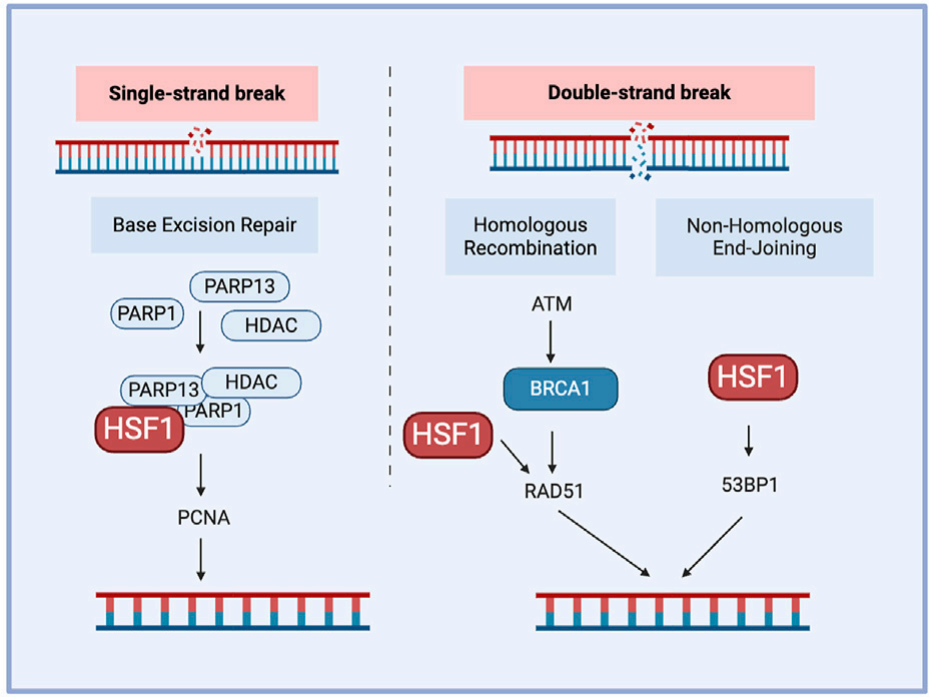
The role of HSF1 in cancer DNA damage repair. HSF1 participates in DNA damage repair by forming interactions with PARP1 and PARP13, facilitating the localization of PARP1 to DNA single-strain break sites and regulating RAD51 or 53BP1 during double-strain break repair. 53BP1. tumor protein p53 binding protein 1; ATM, ataxia-telangiectasia mutated; BRCA1, breast cancer type 1 susceptibility protein; HDAC, histone deacetylase; HSF1, heat shock factor 1; PARP, poly (ADP-ribose) polymerase; PCNA, proliferating cell nuclear antigen; RAD51, RAD51 recombinase.

Single-strand DNA breaks are repaired through the base excision repair pathway, where PARP detects the damage sites and recruits repair factors to them while proliferating cell nuclear antigen (PCNA) acts as a sliding clamp to facilitate the recruitment of DNA polymerases, ensuring efficient repair and genome stability. HSF1 also contributes to DNA damage response by forming a complex with PARP1 and PARP13 and redistributing PARP1 to DNA lesions (Fujimoto et al., 2017). PARP1 contains a C-terminal catalytic domain that helps the synthesis of PAR, and autoPARylation of PARP1 regulates chromatin remodeling and DNA damage repair. Deficiency of HSF1 also reduces the expression of DNA damage repair factors such as RAD51 and 53BP1 and reduces DNA damage repair efficiency. BRCA1 and BRCA2 are DNA damage repair genes; many mutations in these genes result in a dysfunctional DNA damage response. HSF1 deficiency has been shown to reduce the proliferation of mammary tumors having dysfunctional HR due to BRCA1 mutations (Fujimoto et al., 2017). This correlated with HSF1’s crucial role in maintaining the genome integrity as a part of the PSR, contributing to BRCA-mutated tumor cells' addiction to HSF1 (Figure 4). This addiction can be leveraged as a targeted therapy approach, increasing the susceptibility of BRCA-mutated cells to chemotherapy.

Besides, HSF1 can form a complex with DNA damage kinases ATR and CHK1, facilitating p53 phosphorylation in response to DNA damage (Logan et al., 2009). HSF1 activation increases DNA damage repair, contributing to radiation resistance and promoting cancer cell survival under treatment stress in colorectal cancer (Li et al., 2023b). MDM2-mediated ubiquitination of HSF1 under stress conditions affects HSF1 stability, facilitating DNA damage repair processes and potentially contributing to resistance against DNA-damaging treatments (Xiang et al., 2023). Besides, inhibiting HSF1 with KRIBB11 disrupts these pathways, reduces MDM2 and other survival proteins, induces apoptosis, and enhances sensitivity to HSP90 inhibitors, making HSF1 a promising target to overcome DNA damage repair-mediated chemoresistance in adult T-cell leukemia (Ishikawa and Mori, 2023). These findings highlight the potential for targeting HSF1 as a therapeutic approach to overcome DNA damage repair-mediated chemoresistance.

Although it is promising to target HSF1 in DNA damage repair chemoresistance, currently there are gaps in our understanding of HSF1’s role in DNA damage repair involving its dual function in promoting or inhibiting repair pathways, its specific interactions with DNA repair proteins, and its context-specific effects across different cancer types. Addressing these questions could improve our understanding of HSF1 as a potential therapeutic target for developing targeted therapies to overcome chemoresistance.

### 3.4 HSF1 and drug efflux transporters

ATP-binding cassette (ABC) transporters contribute to drug resistance. At the basal level, these transporters export hydrophobic molecules to the outside of cells. However, in cancer cells, the upregulated efflux of drugs through ABC transporters contributes to another potential drug resistance mechanism (Gumilar et al., 2023). ATP-binding cassette sub-family G member 2 (ABCG2), commonly known as breast cancer resistance protein (BCRP), is an important factor in the development of chemoresistance in many malignancies. ABCG2 actively remove a variety of chemotherapeutic drugs out of cancer cells, lowering intracellular drug accumulation and effectiveness (Doyle and Ross, 2003; Noguchi et al., 2014; Mao and Unadkat, 2015). ABCG2 is extensively expressed in some cancer stem cells and its expression is often increased in response to chemotherapy, which helps these cells surviving treatments. Overexpression of ABCG2 has been associated with resistance to many chemotherapeutic treatments, including mitoxantrone, topotecan, and doxorubicin. Targeting ABCG2-mediated drug efflux therefore has emerged as a promising strategy for combating chemoresistance and improving treatment results in cancer patients (Zhang, 2007; Peng et al., 2010; Mo and Zhang, 2012). ABCG1, another member of the ABCG subfamily of ABC transporter, regulates and maintains cellular cholesterol homeostasis and is critical for the survival and function of normal cells. HSF1 overexpression has been seen in melanoma cell lines, contributing to greater drug efflux (Vydra et al., 2013). HSF1 regulates drug resistance through multiple mechanisms. As the mere binding of HSF1 to the HSE element on the ABCG1 gene is insufficient to activate the ABCG1 expression, the detailed mechanism of HSF1 in regulating ABGC1 protein is not understood. However, there are reasonable hypotheses that the ABCG1 activity is post-transcriptionally regulated by the overexpression of HSF1 (Vydra et al., 2013). Interestingly, ABCG1 and ABCG2 are co-expressed in metastatic colon cancer cells, with ABCG1 influencing ABCG2 expression through the modulation of HIF-1α (Namba et al., 2018). This interaction suggests a crosstalk between ABCG1 and ABCG2, implicating HSF1 in regulating drug resistance and tumorigenesis by affecting ABCG2 through ABCG1.

**FIGURE 5:**
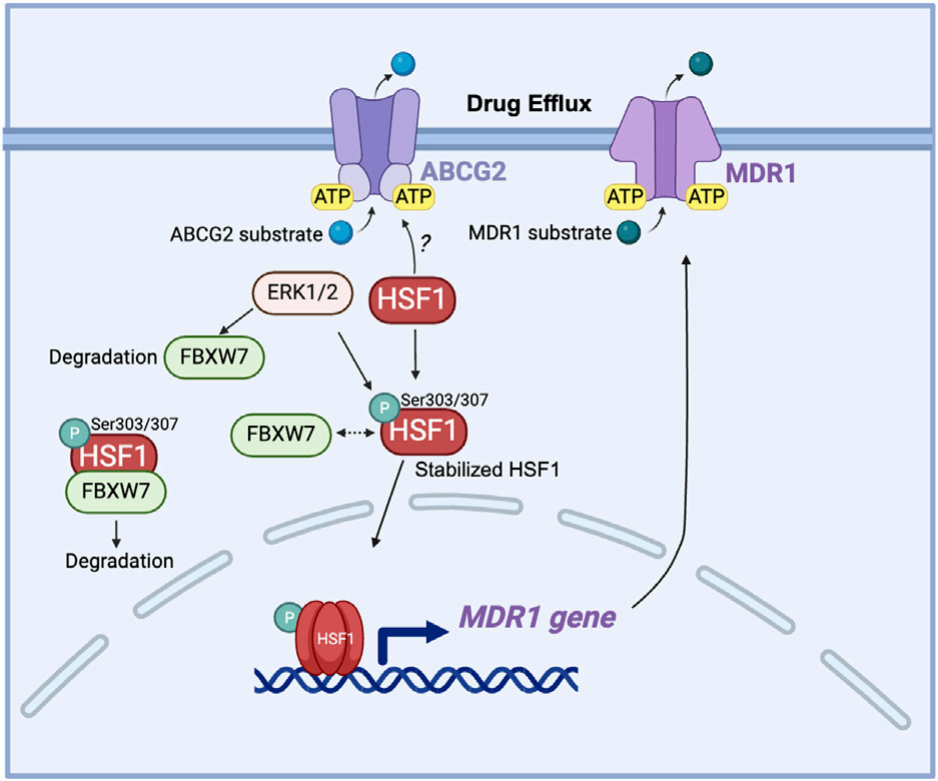
The role of HSF1 in cancer drug efflux. HSF1 upregulates the MDR1 gene expression in drug-resistant cells and is associated with the expression of ABCG2. ERK1/2 phosphorylates FBXW7 at the Thr205 residue, leading to the degradation of FBXW7. This degradation of FBXW7 reduces its ability to ubiquitinate and target HSF1 for proteasomal degradation, resulting in the stabilization of HSF1. The stabilized HSF1 then enhances the transcription of the *MDR1* gene, increasing MDR1 protein expression. Elevated levels of MDR1 contribute to drug resistance by actively pumping chemotherapeutic drugs out of cancer cells, thereby reducing their efficacy. Dashed arrow: dissociation. ABCG2, ATP-binding cassette sub-family G member 2; ATP, adenosine triphosphate; ERK: extracellular signal-regulated kinase; FBXW7, F-box and WD repeat domain-containing 7; HSF1, heat shock factor 1; MDR1, multidrug resistance protein 1 (also known as P-glycoprotein); P, phosphorylation; Ser, serine.

Another key mechanism is that HSF1 acts as the major transcriptional regulator of the multidrug-resistant 1 gene (*MDR1*), which encodes the ABC transporter P-glycoprotein responsible for driving chemoresistance (Krishnamurthy et al., 2012; Gumilar et al., 2023). Overexpression of activated HSF1 increases the MDR1 mRNA level along with enhanced P-glycoprotein cell surface expression (Vilaboa et al., 2000). Interestingly, this function of HSF1 persists even in HSF1 mutants that are unable to increase the transcription of HSP genes, implying that HSF1 may also activate *MDR1* through a non-transcriptional, stress-independent pathway (Tchenio et al., 2006). This finding expands the understanding of HSF1’s role in chemoresistance, which may involve additional, pathways that do not rely solely on stress-induced HSP gene activation. HSF1, through its regulation of HSPs and interaction with SIRT1, contributes to 17-allylamino-17-demethoxygeldanamycin (17-AAG)-mediated chemoresistance in cancer stem-like cells by stabilizing key proteins and enhancing MDR1 drug efflux mechanisms (Kim et al., 2015). F-Box And WD Repeat Domain Containing 7 (FBXW7) has been reported to regulate the stability of nuclear HSF1 by binding to phosphorylated HSF1 at Ser303/307 and leading to HSF1 degradation (Kourtis et al., 2015; Yu et al., 2024). In drug-resistant cells, decreased FBXW7 expression driven by ERK1/2 activation stabilizes phosphorylated HSF1, which in turn enhances the *MDR1* transcription by directly binding to the *MDR1* promoter (Mun et al., 2020). This reveals the post-translational regulation of HSF1 in MDR1-mediated drug resistance (Figure 5).

Targeting HSF1 in conjunction with ABC transporters, particularly ABCG2 and ABCG1, represents a promising strategy for overcoming chemoresistance in various cancers. Inhibiting HSP90 with geldanamycin (GDN) or 17-AAG induces significant apoptotic cell death in glioma cell lines, where ABCG2 shows minimal and ineffective efflux of GDN and 17-AAG (Pastvova et al., 2021). HSP90 inhibitors have been known to induce HSF1 activation (Kijima et al., 2018); however, whether HSF1 regulates ABCG2 stability and expression is still unknown. Furthermore, HSF1 has both transcriptional and non-transcriptional roles in regulating drug efflux pathways, adding complexity to resistance mechanisms but reinforcing its potential as a valuable target for overcoming transporters-mediated resistance, including through MDR1. Further research is needed to elucidate the mechanisms of interaction between HSF1 and ABC transporters to optimize therapeutic strategies in cancer chemoresistance.

### 3.5 HSF1 inhibition in KRAS-mutated cancer

Cancers driven by KRAS mutants often show significant resistance to conventional and targeted chemotherapies, making them particularly challenging to treat (Negri et al., 2022; Singhal et al., 2024). For example, KRAS mutations are known to confer primary and secondary resistance to EGFR-targeted therapies in CRC and NSCLC. This is mainly because that KRAS functions downstream of receptor tyrosine kinases (RTKs), thus, constitutive KRAS activation will activate downstream signalings independently of the upstream RTK activity (Siddiqui and Piperdi, 2010; Zhao et al., 2017; Zhu et al., 2021). To effectively treat KRAS-driven tumors and to overcome chemoresistance conferred by KRAS mutants, effective inhibition of KRAS mutants would be ideal. Despite four decades of intensive research, targeting KRAS mutations directly remains challenging due to the protein’s structural characteristics, which lack deep binding pockets, limiting the efficacy of traditional small-molecule inhibitors (Huang et al., 2021; Wadood et al., 2022; Zhu et al., 2022). Additionally, the mechanisms by which KRAS mutations confer chemoresistance, including interactions with downstream signaling pathways such as HSF1-mediated stress responses, are not fully understood.

An exciting recent advance in inhibiting KRAS mutants is the approval of KRAS(G12C)-mutant inhibitors for the clinical use. Both adagrasib and sotorasib are developed to target the KRAS(G12C)-mutant by covalently binding to the mutant cysteine residue in KRAS(G12C), leading to irreversible inhibition of the KRAS activity and blocking KRAS downstream signaling pathways essential for cancer cell proliferation. Both adagrasib and sotorasib have been approved explicitly for treating KRAS(G12C)-mutant NSCLC (Huang et al., 2021; Janne et al., 2022). However, G12C is only one of many mutations that having been found in KRAS in tumors and acquired resistance to KRAS(G12C) inhibitors has been observed. KRAS mutant tumors treated with KRAS inhibitors often develop resistance to these inhibitors through acquired *KRAS* alternations, *MET* amplification or oncogenic *BRAF* gene fusions and mutations. These tumors may also undergo phenotypic changes, such as epithelial-mesenchymal transition (EMT), or adopt survival mechanisms like metabolic rewiring and autophagy (Awad et al., 2021; Yaeger et al., 2023). For example, although adagrasib can reverse multidrug resistance mediated by the MDR1 transporter (Zhang et al., 2022), the resistance to adagrasib has emerged through heterogeneous subclonal mutations in RAS-MAPK pathway components (e.g., NRAS, BRAF, and MAP2K1) that enable NSCLC tumor cells to reactivate MAPK signaling and bypass KRAS(G12C) inhibition (Tanaka et al., 2021). In order to obtain more durable responses to KRAS inhibitors, researchers are exploring combination therapies, such as pairing KRAS inhibitors with MEK or SHP2 inhibitors or with immune checkpoint inhibitors (Khan and O'Bryan, 2021; Hegazi et al., 2024). Thus, targeting KRAS for cancer treatment remains challenging, particularly in overcoming chemoresistance.

HSF1 is a downstream effector of the RAS-MEK pathway (Tang et al., 2015; Dai and Sampson, 2016) and KRAS-mutant cancers, due to their elevated levels of cellular stress resulting from rapid proliferation and metabolic demands, have a higher dependence on HSF1. Therefore, HSF1 represents a promising target for KRAS-mutant cancers, potentially addressing some resistance mechanisms by disrupting cancer cell stress responses. By targeting HSF1, it may be possible to exploit this stress vulnerability, making KRAS-mutant cancer cells more susceptible to therapeutic intervention. Targeting HSF1 in KRAS-mutant cancers, such as PDAC and NSCLC, where chemoresistance is common and therapeutic options are limited could open new avenues for treatment. By specifically disrupting the HSF1-mediated stress response in these tumors, HSF1 inhibitors may provide a novel approach to sensitize these cancers to existing therapies and improve clinical outcomes.

### 3.6 Challenges and opportunities in targeting HSF1 for cancer therapy

Several small molecules targeting HSF1 have been synthesized and their effects have been tested, primarily in *in vitro* and *in vivo* preclinical models. However, because HSF1 is a transcription factor and lacks clearly targetable sites, developing drugs that specifically target HSF1 remains highly challenging (Gumilar et al., 2023). Several HSF1 inhibitors have been discussed broadly (Dong et al., 2019; Cyran and Zhitkovich, 2022). Most current inhibitors target HSF1 indirectly and suffer from limited specificity and potency. Additionally, HSF1’s role in tumorigenesis is complex, involving multiple signaling pathways that vary across different cancer types. Although several potential HSF1 inhibitors have been identified, we will mainly discuss current small molecules that directly interact with HSF1 in this review.

KRIBB11 is a small molecule known to directly associate with HSF1, disrupting its functional activity by preventing the recruitment of pTEFB on the promoter region of HSP70, as seen in a colorectal carcinoma cell line (Yoon et al., 2011). This blocks the transcription of *HSP70* and *HSP27* and the downstream stress response while inducing growth arrest and triggering caspase-dependent apoptosis. For example, in lung cancer, KRIBB11 induces apoptosis by reducing the level of Mcl-1, an anti-apoptotic protein (Kang et al., 2017). KRIBB11 induces apoptosis and cell cycle arrest in NSCLC, especially in combination therapies, by inhibiting the PI3K/AKT pathway, increasing ROS, and activating DNA damage responses (Zhang et al., 2024). In another study of NSCLC, KRIBB11 is shown to reduce drug resistance potentially associated with EMT by downregulating EMT-associated proteins, such as N-cadherin and vimentin, and EGFR, along with other key signaling molecules (Shibue and Weinberg, 2017; Lee et al., 2021). Although KRIBB11 directly associates with HSF1, it may also interact with other proteins or pathways, leading to off-target effects that lead to toxicities and narrowing the therapeutic window, thus complicating its clinical application.

DTHIB binds HSF1 directly and degrades HSF1 in the nucleus, thereby decreasing its nuclear activity and ultimately lowering its transcriptional activity in prostate cancer (Dong et al., 2020). DTHIB induces the degradation of HSF1 through the proteasome and the E3 ligase component FBXW7. Additionally, DTHIB effectively attenuated tumor growth in four therapy-resistant prostate cancer animal models, including a neuroendocrine prostate cancer model, where it caused profound tumor regression (Dong et al., 2019; Dong et al., 2020). However, further development and optimization are necessary to assess its efficacy in KRAS mutant cancers before advancing to clinical trials.

NXP800 (CCT361814) is currently the only inhibitor targeting the HSF1 pathway that has advanced to clinical trials, it is now in phase Ib clinical trial in platinum-resistant, ARID1a-mutated ovarian cancer (NCT05226507) (Cheeseman et al., 2017; clinicaltrials.gov 2021; Pasqua et al., 2023). This marks a significant milestone in HSF1-targeted cancer therapy. NXP800 has been shown to inhibit HSF1 transcriptional activity (Menezes et al., 2017; Workman et al., 2022). NXP800 inhibits HSF1 indirectly by activating the integrated stress response (ISR) through the general control nonderepressible 2 (GCN2), Activation of GCN2 leads to phosphorylation of eukaryotic translation initiation factor 2 α (eIF2α), which reduces global protein synthesis and selectively increases the translation of stress-responsive genes like activating transcription factor 4 (ATF4). Elevated ATF4 levels subsequently inhibit HSF1 activation, thereby diminishing the expression of HSF1-regulated genes This mechanism has been observed in human carcinoma cell lines and tumor xenograft models, where NXP800-induced ISR activation resulted in decreased HSF1 activity and reduced tumor cell proliferation (Cheeseman et al., 2017; Pasqua et al., 2023). NXP800-mediated inhibition of HSF1 induces sustained cellular stress, ultimately triggering programmed cell death. This therapeutic mechanism is particularly effective in cancers that heavily rely on HSF1 for protection against stress-induced damage, as it weakens the cells’ defenses against therapeutic intervention. However, further extensive research is necessary to optimize this therapeutic approach, including investigations into optimal dosing strategies, potential synergistic effects with various chemotherapy agents, and a deeper understanding of the molecular pathways by which HSF1 inhibition impacts cancer cell survival and treatment resistance.

Technological advancements are revolutionizing the creation of HSF1 inhibitors to address chemoresistance. Proteolysis-targeting chimeras, or PROTACs, offer a strong alternative by completely degrading HSF1 rather than just inhibiting it, which has the potential to reduce the possibility of resistance. A proof-of-concept study has demonstrated the feasibility of HSF1 degradation using a bifunctional PROTAC molecular that leverages KRIBB11-mediated binding and E3 ligase-mediated proteolysis (Sharma et al., 2022). Although this study has demonstrated the utility of the PROTAC approach for HSF1 inhibition, the effect of this approach on chemoresistance remains to be evaluated. Besides, an RNA aptamer that binds specifically and tightly to the DNA-binding domain of HSF1 has been developed and demonstrated to be able to block HSF1 from binding to DNA when delivered using a synthetic gene and strong promoter (Salamanca et al., 2011). Overall, the study highlights aptamers’ potential for precise inhibition of HSF1 in cancer while minimizing off-target effects, providing a novel strategy to suppress cancer cell growth and survival by blocking HSF1’s DNA-binding activity (Salamanca et al., 2014). Recently, nanoparticle drug delivery systems have demonstrated effective in enhancing the potency of HSF1 inhibitors by targeting them directly to tumor tissues. This approach reduces off-target side effects and maintains reliable treatment drug concentration. For example, one study shows that functionalized nanomaterials can efficiently transport small-molecule HSP inhibitors to tumor locations, boosting the effectiveness of both photothermal and photodynamic therapies (Premji et al., 2024). In addition, studies on hybrid nanoparticles enhanced with hyaluronic acid for delivering an HSP90 inhibitor emphasize their potential in targeted cancer therapy by enhancing drug delivery efficiency and specificity (Pan et al., 2021). Another study has developed aqueous bovine serum albumin nanoparticles for controlled delivery of the Hsp90 inhibitor luminespib. Through *in vitro* characterization and evaluations, this approach has demonstrated its potential as a nanoformulation for pancreatic and breast cancer therapy (K Rochani et al., 2020). Using advanced patient-derived models like organoids and xenografts allows for the assessment of HSF1 inhibitors in settings that closely resemble human tumors. This method results in more reliable predictions about their effectiveness and possible resistance (Yang et al., 2018). In summary, these advancements are driving the development of more effective HSF1-targeted therapies to tackle the issue of chemoresistance in cancer treatment specifically.

## 4 Conclusion and perspective

This review explores mechanisms of the HSF1’s role in the proteotoxic stress response and its function during chemotherapy exposure. HSF1 regulates multiple pathways that contribute to chemoresistance throughout tumorigenesis. Because HSF1 is active in normal and cancerous cells, complete inhibition of HSF1 as a cancer treatment approach remains challenging. Among autophagy, apoptosis resistance, DNA damage repair, and drug efflux mechanisms, autophagy is often considered the key downstream pathway regulated by HSF1 in the context of chemotherapy resistance. HSF1-mediated autophagy enables cancer cells to manage cellular stress by degrading and recycling damaged proteins and organelles, supporting cell survival under chemotherapy-induced stress. This autophagy-driven survival mechanism is crucial, allowing cancer cells to resist apoptosis and maintain functionality despite therapeutic interventions. Therefore, while drug efflux, apoptosis resistance, and DNA damage repair are important, autophagy is likely the primary downstream pathway through which HSF1 exerts its protective effects against chemotherapy. Given the high expression of HSF1 in various cancer types and its role in chemoresistance, inhibiting HSF1 may offer a promising therapeutic strategy to counteract chemoresistance. This strategy could potentially disrupt the mechanisms that enable cancer cells to resist chemotherapy, enhancing the efficacy of treatment.
